# Reduced Parasite Burden in Children with Falciparum Malaria and Bacteremia Coinfections: Role of Mediators of Inflammation

**DOI:** 10.1155/2016/4286576

**Published:** 2016-06-22

**Authors:** Gregory C. Davenport, James B. Hittner, Vincent Otieno, Zachary Karim, Harshini Mukundan, Paul W. Fenimore, Nicolas W. Hengartner, Benjamin H. McMahon, Prakasha Kempaiah, John M. Ong'echa, Douglas J. Perkins

**Affiliations:** ^1^Department of Infectious Diseases and Microbiology, Graduate School of Public Health, University of Pittsburgh, Pittsburgh, PA, USA; ^2^Center for Global Health, Department of Internal Medicine, Division of Infectious Diseases, School of Medicine, University of New Mexico, Albuquerque, NM 87131, USA; ^3^Department of Psychology, College of Charleston, Charleston, SC, USA; ^4^University of New Mexico/KEMRI Laboratories of Parasitic and Viral Diseases, Centre for Global Health Research, Kenya Medical Research Institute, Kisumu, Kenya; ^5^Chemistry Division, Los Alamos National Laboratory, Los Alamos, NM, USA; ^6^Theoretical Biology Group, Theoretical Division, Los Alamos National Laboratory, Los Alamos, NM, USA

## Abstract

Bacteremia and malaria coinfection is a common and life-threatening condition in children residing in sub-Saharan Africa. We previously showed that coinfection with Gram negative (G[−]) enteric Bacilli and* Plasmodium falciparum* (*Pf*[+]) was associated with reduced high-density parasitemia (HDP, >10,000 parasites/*μ*L), enhanced respiratory distress, and severe anemia. Since inflammatory mediators are largely unexplored in such coinfections, circulating cytokines were determined in four groups of children (*n* = 206, aged <3 yrs): healthy;* Pf*[+] alone; G[−] coinfected; and G[+] coinfected.* Staphylococcus aureus* and non-Typhi* Salmonella* were the most frequently isolated G[+] and G[−] organisms, respectively. Coinfected children, particularly those with G[−] pathogens, had lower parasite burden (peripheral and geometric mean parasitemia and HDP). In addition, both coinfected groups had increased IL-4, IL-5, IL-7, IL-12, IL-15, IL-17, IFN-*γ*, and IFN-*α* and decreased TNF-*α* relative to malaria alone. Children with G[−] coinfection had higher IL-1*β* and IL-1Ra and lower IL-10 than the* Pf*[+] group and higher IFN-*γ* than the G[+] group. To determine how the immune response to malaria regulates parasitemia, cytokine production was investigated with a multiple mediation model. Cytokines with the greatest mediational impact on parasitemia were IL-4, IL-10, IL-12, and IFN-*γ*. Results here suggest that enhanced immune activation, especially in G[−] coinfected children, acts to reduce malaria parasite burden.

## 1. Introduction

Kenya is one of WHO African-region countries that bears the greatest global yearly burden of malaria, affecting primarily children less than five years of age and pregnant women [1]. Western Kenya is a holoendemic area of* Plasmodium falciparum* transmission which leads to early and chronic exposure of children to the malaria parasite and, consequently, a high rate of severe malaria anemia (SMA, Hb < 5.0 g/dL) [2]. In addition to malaria, other diseases such as bacteremia, human immunodeficiency virus (HIV)-1, and hookworm can contribute to childhood anemia [3–5].

Bacteremia in children is a worldwide problem and concern but is particularly dangerous in tropical and subtropical regions where children are concomitantly compromised by* P. falciparum* and HIV-1 infections. Bacteremia caused by non-Typhi* Salmonella* (NTS) is also a common finding among young children and those infected with HIV-1 in sub-Saharan Africa [5, 6]. Many studies have associated NTS bacteremia with malnutrition [7–10], which is nearly ubiquitous in these regions (reviewed in [11]). We have recently reported an 11.8% bacteremia rate in our study population in Siaya, Kenya, with 50% of the isolates comprised of NTS, followed by* Staphylococcus aureus* (32%) as the predominant Gram positive (G[+]) cocci [5]. This study also demonstrated that G[−] organisms were associated with reduced high-density parasitemia (HDP, >10,000 parasites/*μ*L), enhanced respiratory distress, and SMA [5].

The early phase of the immune response to both malaria and bacteremia monoinfections are characterized by the release of greatly elevated levels of cytokines such as TNF-*α*, IL-1, and IL-6 [12–16]. Each of the diseases elicits a potent type 1 inflammatory response (e.g., TNF-*α* and IFN-*γ*), which, if not counterregulated by type 2 immunity (e.g., IL-10), can cause significant short- and long-term adverse clinical outcomes and death if not properly controlled [14–18]. Early production of TNF-*α*, IFN-*γ*, IL-1, IL-6, and IL-12 allows for effective and rapid parasite clearance in humans with falciparum malaria [14, 19–21]. Once parasite replication has been controlled, we and others have shown that anti-inflammatory cytokines, such as IL-10 and TGF-*β*, are required for prevention of host damage and progression towards severe malaria [22–24]. However, research from our laboratory has also shown that IL-12 is significantly reduced in children with SMA by an IL-10-dependent mechanism [25]. Reduced IL-12 is also associated with an increased risk of neonatal sepsis [26], whereas high levels of IL-10 are associated with septic shock and have a high predictive value on clinical outcomes [27, 28]. Similarly, IL-12 can also be antagonized by high levels of TNF-*α* [29], which is a hallmark of malaria infections, as well as Gram negative (G[−]) sepsis [30]. Thus, severe malaria appears to be a disease defined by an inappropriate response in* magnitude* and* timing*, rather than an inappropriate response in the* type* of inflammatory mediators released, much like bacterial sepsis.

Since the inflammatory milieu in children with malaria and bacteremia coinfection is largely undefined, this study examined pro- and anti-inflammatory cytokine responses in Kenyan children living in a holoendemic region for* P. falciparum* transmission in which bacteremia is a frequent complicating feature of malaria. The primary hypothesis tested in this study was that mediators of inflammation are important determinants of parasite burden in children with malaria and bacteremia coinfections. Characterization of cytokine profiles in children with common endemic comorbidities is important step for the future design of diagnostic strategies based on host biomarkers.

## 2. Methods

### 2.1. Study Participants

Children 3–36 months of age (*n* = 206) with and without febrile illness were recruited at Siaya County Referral Hospital, western Kenya, during their first hospital contact for treatment of febrile illness. Prior presentation at the hospital was determined through existing medical records. Children presented in the study are a subset of children from a larger cohort (*n* = 898) designed to investigate the pathogenesis of severe malarial anemia and related comorbidities. Siaya County is a holoendemic* P. falciparum* transmission area [31]. None of the children had cerebral malaria or nonfalciparum malarial infections as they are rare occurrences in this region. A detailed description of the study area and population can be found in our previous publication [3].

Children with malaria parasites were divided into three groups: malaria only (parasitemic,* Pf*[+], *n* = 156); Gram negative bacteremia (G[−] coinfected, *n* = 22); and Gram positive bacteremia (G[+] voinfected, *n* = 14). In addition, a healthy control group (*n* = 14) composed of afebrile, uninfected children with Hb > 11.0 g/dL was included for comparison. Healthy children were recruited at the hospital during presentation for their childhood vaccinations. HIV-1 status was determined using the serological antibody tests Uni-Gold*™* (Trinity Biotech) and Determine® (Abbott Laboratories) and HIV-1 DNA PCR analysis was performed according to our previously published methods [32]. Children found to be either exposed to or infected with HIV-1 were excluded from these analyses, since our previous study demonstrated significant inflammatory mediator perturbations in children with these comorbidities [32], which detract from the specific inflammatory profiles targeted in the current study. Pre- and posttest HIV counseling were provided for the parents/guardians of all participants.

Children were also stratified into SMA (Hb < 5.0 g/dL) to determine if severe anemia differed among the groups [33]. Children were treated according to Ministry of Health, Kenya, guidelines and all samples and clinical measures were obtained prior to any treatment intervention at the hospital. Written informed consent was obtained from the participants' parents/guardians. Approval for the study was granted by the University of Pittsburgh, University of New Mexico, and the Kenya Medical Research Institute (KEMRI).

### 2.2. Blood Culture

Blood culture was performed upon enrollment on every study participant, regardless of clinical presentation. The phlebotomy site and Wampole*™* Isostat® Pediatric 1.5 tube stopper were cleaned with 70% ethanol prior to venipuncture. Blood was processed according to the manufacturer's instructions and plated on standard microbiological media. Briefly, the entire Isostat supernatant was plated on Chocolate Agar and incubated at 35°C in 5% CO_2_. If no growth was detected after 18–24 hrs of incubation, an additional incubation period of four days was employed with daily inspection for growth. Unique colonies from subculture were identified using standard microbiological and biochemical techniques (i.e., Gram stain, colony characteristics, API biochemical panels, and agglutination tests). Specifically, G[−] isolates were identified using API® 20E for oxidase negative Enterobacteriaceae or API20NE for oxidase positive organisms (BioMérieux, Inc.), while G[+] organisms were identified using various biochemical techniques (i.e., catalase, hemolysis patterns, serologic grouping, and coagulase test). Gram positive Bacilli and Coagulase Negative Staphylococcus (CoNS) were considered laboratory/skin contaminants and not included in our analyses.

### 2.3. Laboratory Measurements

Asexual malaria trophozoites were determined according to previous methods [34]. Complete blood counts were performed using a Beckman Coulter^©^A^c^-T diff2*™* (Beckman Coulter, Inc.) on blood obtained prior to administration of antimalarial, antibiotics, and/or antipyretics.

### 2.4. Multiplex Assay

Venous blood samples were centrifuged and plasma was separated immediately, aliquoted, and stored at −70°C. Samples were then thawed and clarified by centrifugation (14,000 rpm for 10 min) immediately prior to assaying. Inflammatory mediator levels were determined by the Cytokine 25-Plex Antibody Bead Kit, Human (BioSource*™* International) according to the manufacturer's instructions. Plates were read on a Luminex 100*™* system (Luminex Corporation) and analyzed using the Bio-Plex Manager Software (Bio-Rad Laboratories). Analyte detection limits were 3.0 pg/mL (IL-5 and IL-6); 5.0 pg/mL (IL-1Ra, IL-3, IL-4, IL-10, and IFN-*γ*); 6.0 pg/mL (IL-2); 10.0 pg/mL (IL-7, IL-13, IL-15, IL-17, and TNF-*α*); and 15.0 pg/mL (IL-1*β*, IL-12p40/p70, and IFN-*α*).

### 2.5. Statistical Analyses

Data were analyzed using SPSS (version 15.0). Clinical, demographic, hematological, and immunological measures were compared across groups by either ANOVA or Kruskal-Wallis (K-W) tests, followed by pair-wise post hoc comparison with Student's *t*-test or Mann-Whitney *U* test, respectively. Pearson's Chi Square (*χ* ^2^) and Fisher's exact test were used for proportion comparison. In the event that the cell sum of categorical analyses was less than 20, as in the mortality data, generalized Fisher's exact test was performed in R version 3.3.2. Statistical significance was set at *P* ≤ 0.05. A multiple mediation model was performed to determine the mediators of inflammation responsible for decreased parasitemia in coinfected children. The SPSS macroprogram (indirect.sps) and details for its use are located on the following website: http://afhayes.com/spss-sas-and-mplus-macros-and-code.html [35].

## 3. Results

### 3.1. Distribution of Bacterial Isolates

The two predominant isolates in the study were* Staphylococcus aureus* (36.1%) and* Salmonella enterica* ssp.* enterica* ser. Typhimurium (44.4%), with NTS comprising 52.8% of the overall isolates (Table 1). Children included in this study were free of other nonbacterial coinfections (i.e., HIV-1 and hookworm) and had the following bacterial isolate distribution: 61.2% G[−] (*S. enterica* ssp.* enterica* ser. Typhimurium,* S. enterica* ssp.* enterica* ser. Enteritidis,* Salmonella enterica* ssp.* enterica* ser. Arizonae,* Pseudomonas aeruginosa*,* Escherichia coli*, and* Acinetobacter* sp.) and 38.8% G[+] (*S. aureus* and* Enterococcus* sp.) organisms.

### 3.2. Demographic, Clinical, and Hematological Characteristics

Children with falciparum malaria were stratified into three categories: malaria alone (*Pf*[+], *n* = 156); G[−] coinfected (*n* = 22); and G[+] coinfected (*n* = 14) (Table 2). A healthy control group was included for comparison (*n* = 14) but excluded from the omnibus statistical comparisons to prevent unfairly skewing the results. Study participant demographics and clinical characteristics are listed in Table 2. The G[−] and G[+] coinfected groups had 38.5% and 28.6% more females than males, respectively (*P* = 0.041). However, age, axillary temperature, and glucose were not statistically different across the groups. Hematologically, there were significant intergroup differences in white blood cell counts (WBC, *P* = 0.047), absolute granulocyte counts (*P* = 0.010), and mean corpuscular hemoglobin concentrations (MCHC, *P* = 0.003) but comparable hemoglobin (Hb, *P* = 0.594), hematocrit (*P* = 0.793), and RBC counts (*P* = 0.642). Post hoc testing revealed that, relative to the* Pf*[+] and G[−] coinfected groups, the G[+] coinfected group had lower WBC counts (*P* = 0.022 and *P* = 0.026, resp.) and granulocyte counts (*P* = 0.003 and *P* = 0.007, resp.), while the MCHC of both the G[−] and G[+] coinfected groups (*P* = 0.008 and *P* = 0.016, resp.) were lower than those of the* Pf*[+] group. In addition, the red cell distribution width (RDW) approached statistical significance, with higher values in coinfected children, potentially indicating that either they were recovering from greater anemia or they have a more robust erythropoietic response (*P* = 0.053). Although we previously found that G[−] coinfection in the same cohort of children was associated with significantly greater severe anemia (Hb < 5.0 g/dL) [5], the proportion of severe anemia did not differ across the malaria-infected groups with and without coinfection (*P* = 0.562). Previous reports have suggested links between glucose-6-phosphate dehydrogenase (G6PD) deficiency and altered cytokine expression [36], as well as increased susceptibility to bacteremia in those carrying sickle cell alleles [37]. However, we noted no significant differences between the three parasitemic groups for either G6PD deficiency (*χ* ^2^ = 2.505, *P* = 0.644) or sickle cell trait/disease (*χ* ^2^ = 0.158, *P* = 0.924). Consistent with our previous study, showing higher mortality in malaria-infected children with G[−] coinfection [5], the highest mortality was observed in the G[−] coinfected group but did not significantly differ across the groups (*P* = 0.459).

### 3.3. Parasitological Indices

**Figure 1:**
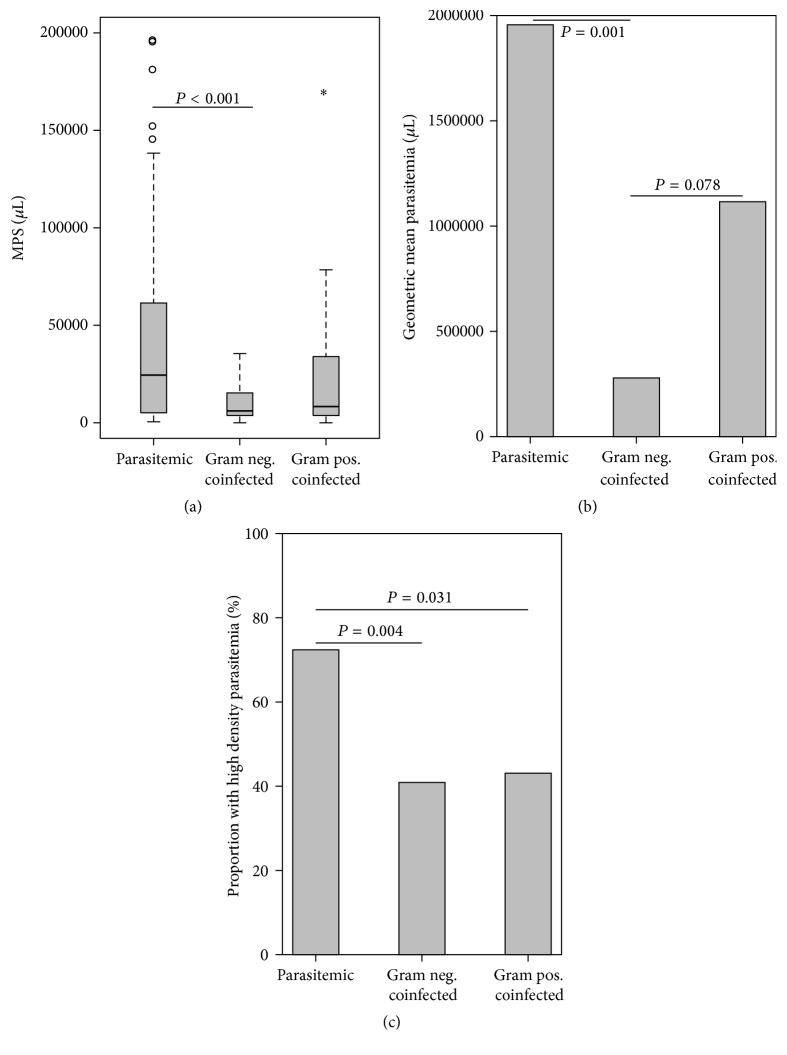
Parasitologic indices. Data are presented as box plots with whiskers and outliers. The box represents the interquartile range, while the whiskers represent the 10th and 90th percentiles. The line across the box indicates the median value, open circles (°) represent extremes, and asterisks (*∗*) depict outliers. Differences between groups were determined using Mann-Whitney *U* test. (a) MPS [malaria parasites] (Kruskal-Wallis [K-W] test, *P* = 0.001). (b) Geometric mean parasitemia (ANOVA, *P* = 0.011). (c) Proportion of high-density parasitemia (%) (K-W test, *P* = 0.002).

Comparison of the three groups showed a significant difference in parasitemia densities (Kruskal-Wallis [K-W], *P* = 0.001; Figure 1(a)). Pair-wise comparison revealed that children with G[−] coinfections (6,461 parasites [MPS]/*μ*L), but not those with G[+] coinfection (8,704 MPS/*μ*L, *P* = 0.217), had significantly lower parasite densities relative to the* Pf*[+] group (25,619 MPS/*μ*L, *P* < 0.001; Figure 1(a)). In addition, the geometric mean parasitemia was greater in children with malaria alone relative to those with G[−] coinfection (*P* = 0.001; Figure 1(b)). Similarly, the* Pf*[+] group had a greater percentage (72.4%) of children with high-density parasitemia (HDP, >10,000 parasites/*μ*L; K-W *P* = 0.002), when compared to either the G[−] (40.9%, *P* = 0.004) or the G[+] (42.9%, *P* = 0.031) coinfected groups (Figure 1(c)). Thus, coinfection, particularly with G[−] organisms, is associated with reduced parasite burden.

### 3.4. Proinflammatory Cytokine Production

**Figure 2:**
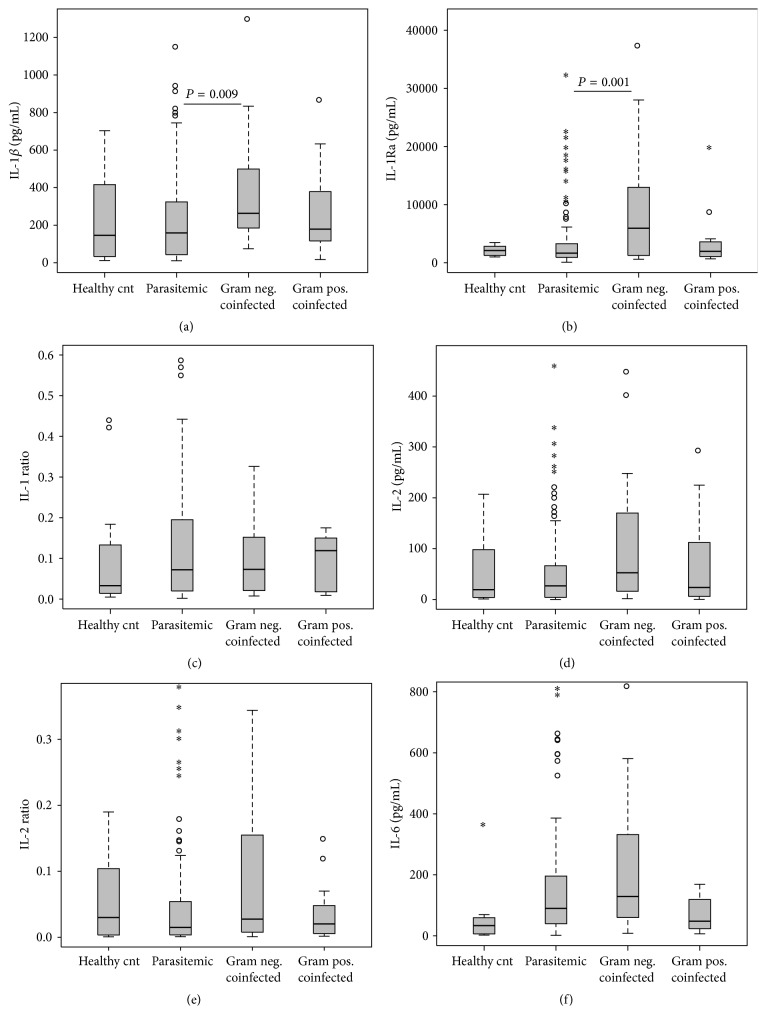
Proinflammatory mediator profiles. Data are presented as box plots with whiskers and outliers. The box represents the interquartile range, while the whiskers represent the 10th and 90th percentiles. The line across the box indicates the median value, open circles (°) represent extremes, and asterisks (*∗*) depict outliers. Pair-wise comparisons between groups were conducted with Mann-Whitney *U* test. All units are in pg/mL. (a) IL-1*β* (Kruskal-Wallis [K-W], test *P* = 0.027). (b) IL-1Ra (K-W test, *P* = 0.005). (c) IL-1 Ratio (K-W, test *P* = 0.854). (d) IL-2 (K-W test, *P* = 0.068). (e) IL-2 Ratio (K-W test, *P* = 0.575). (f) IL-6 (K-W test, *P* = 0.115).

Cytokine dysregulation is thought to be central to the pathological processes of severe malaria and sepsis (reviewed in [38]). We, [3, 23, 25, 39–43] and others [44], have demonstrated that childhood malaria and the subsequent anemia that ensues are exacerbated by dysregulation of pro- and anti-inflammatory cytokines, the same inflammatory mediators that are responsible for controlling parasitemia [14, 19–21]. However, since the circulating inflammatory mediator profile in malaria and bacteremia coinfected children has not been reported, circulating levels of cytokines were determined with a multiplex bead array assay. These experiments revealed that IL-1*β* and its receptor antagonist (IL-1Ra) both had significant intergroup differences (*P* = 0.027 and *P* = 0.005, resp.). Pair-wise analysis revealed significantly elevated levels of IL-1*β* and IL-1Ra in children with G[−] coinfection (Figure 2(a), *P* = 0.009; Figure 2(b), *P* = 0.001, resp.), compared to* Pf*[+]-only children. However, the ratio of these mediators did not differ across the groups (IL-1 : IL-1Ra, *P* = 0.854; Figure 2(c)), nor did levels of IL-2 (*P* = 0.068; Figure 2(d)), the IL-2 : IL-2R ratio (*P* = 0.226; Figure 2(e)), or IL-6 (*P* = 0.115; Figure 2(f)).

**Figure 3:**
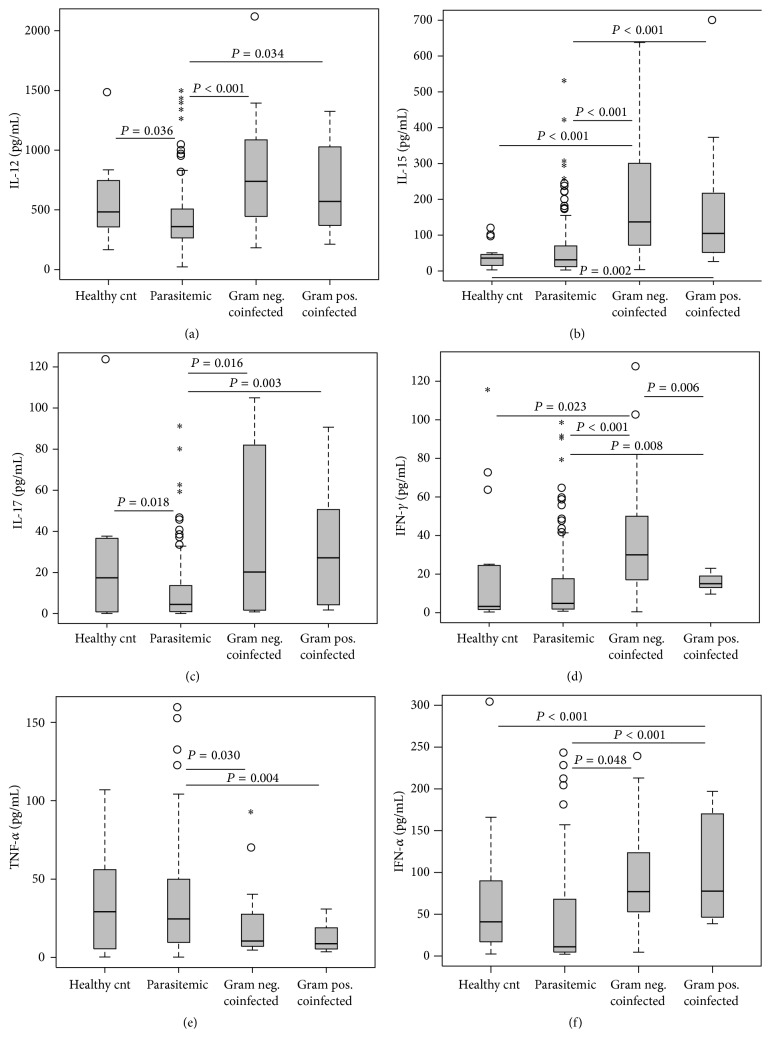
Proinflammatory mediator profiles. Data are presented as box plots with whiskers and outliers. The box represents the interquartile range, while the whiskers represent the 10th and 90th percentiles. The line across the box indicates the median value, open circles (°) represent extremes, and asterisks (*∗*) depict outliers. Pair-wise comparisons between groups were conducted with Mann-Whitney *U* test. All units are in pg/mL. (a) IL-12 (Kruskal-Wallis [K-W] test, *P* < 0.001). (b) IL-15 (K-W test, *P* < 0.001). (c) IL-17 (K-W test, *P* = 0.002). (d) IFN-*γ* (K-W test, *P* < 0.001). (e) TNF-*α* (K-W test, *P* = 0.003). (f) IFN-*α* (K-W test, *P* < 0.001).

As shown in Figure 3(a), circulating IL-12 differed significantly across the groups (*P* < 0.001) with the highest concentrations in the G[−] coinfected group (*P* < 0.001 versus* Pf*[+]), followed by the G[+] coinfected group (*P* = 0.034 versus* Pf*[+]). The healthy controls also had greater IL-12 values than the* Pf*[+] children (*P* = 0.036). An identical pattern and magnitude were observed in the IL-15 levels (K-W, *P* < 0.001; Figure 3(b)), with the G[−] and G[+] groups both having higher levels than the* Pf*[+] children (*P* < 0.001 and *P* < 0.001, resp.) and healthy controls (*P* < 0.001 and *P* = 0.002, resp.). There were similar levels of IL-17 (K-W, *P* = 0.002; Figure 3(c)) in the healthy controls, G[−] coinfected, and G[+] coinfected groups, compared to the significantly lower levels in the* Pf*[+] group (*P* = 0.018, *P* = 0.016, and *P* = 0.003, resp.). IFN-*γ* levels (K-W, *P* < 0.001; Figure 3(d)) were similar to the patterns seen in IL-12 and IL-15, with the G[−] group having the highest levels, relative to the moderate levels in the G[+] group (*P* = 0.006), and negligible levels were detected in the* Pf*[+] children (*P* < 0.001) and healthy controls (*P* = 0.023). The G[+] coinfected group also had higher levels of IFN-*γ* relative to the malaria-only group (*P* = 0.008). TNF-*α* levels were highest in the healthy control group (K-W, *P* = 0.003; Figure 3(e)), followed by the* Pf*[+] children and the two coinfected groups, while the G[−] (*P* = 0.030) and G[+] (*P* = 0.004) coinfected groups' concentrations were significantly lower than the* Pf*[+] group. As was observed with the IL-12 and IL-17 levels, the* Pf*[+] group had significantly depressed levels of IFN-*α* (K-W, *P* < 0.001; Figure 3(f)) compared to the G[−] (*P* < 0.001) and G[+] (*P* < 0.001) coinfected groups (Figure 3(f)).

### 3.5. Anti-Inflammatory Cytokine Production

**Figure 4:**
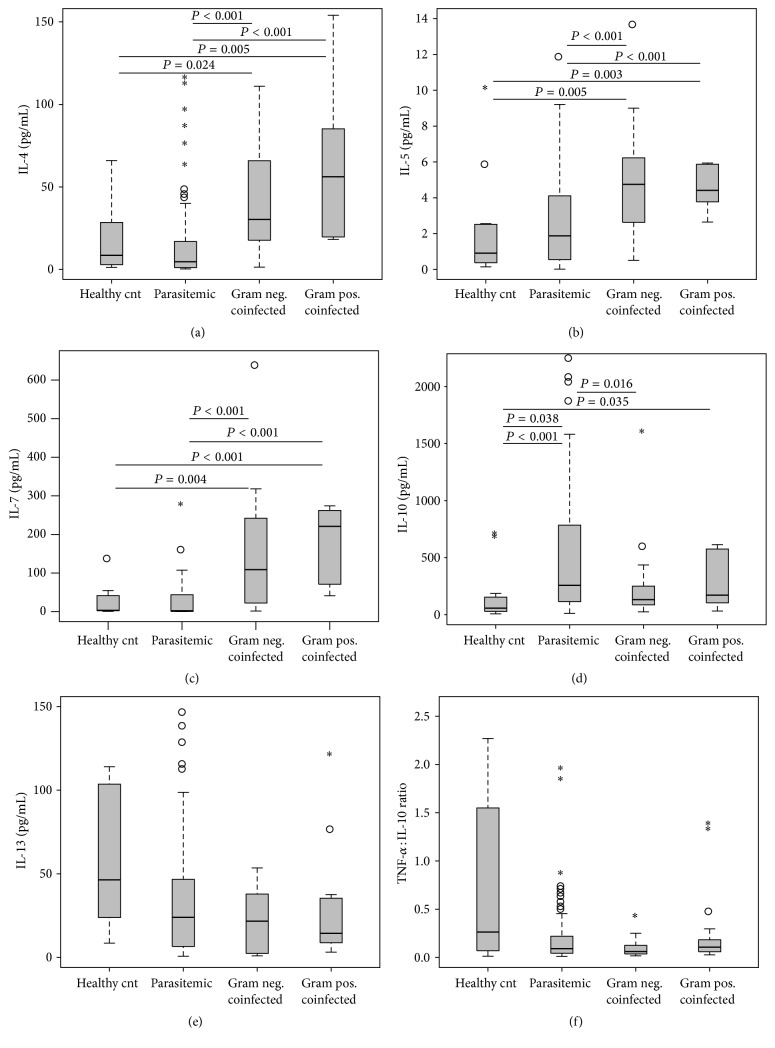
Anti-inflammatory mediator profiles. Data are presented as box plots with whiskers and outliers. The box represents the interquartile range, while the whiskers represent the 10th and 90th percentiles. The line across the box indicates the median value, open circles (°) represent extremes, and asterisks (*∗*) depict outliers. Pair-wise comparisons between groups were conducted with Mann-Whitney *U* test. All units are in pg/mL. (a) IL-4 (Kruskal-Wallis [K-W], test *P* < 0.001). (b) IL-5 (K-W test, *P* < 0.001). (c) IL-7 (K-W test, *P* < 0.001). (d) IL-10 (K-W test, *P* = 0.042). (e) IL-13 (K-W test, *P* = 0.366). (f) TNF-*α* : IL-10 ratio (K-W test, *P* = 0.329).

The anti-inflammatory cytokines, specifically IL-10, are known to play a key role in abrogating a robust initial proinflammatory response to malaria, as well as recovery from anemia [45]. However, we have previously shown that phagocytized* Pf*Hz promotes overproduction of IL-10,in turn suppressing IL-12, thereby exacerbating anemia and slowing parasite clearance [40]. Upon examination of the anti-inflammatory cytokines, we observed similar patterns to those observed for the proinflammatory molecules in which coinfected children had the highest levels relative to the monoinfected* Pf*[+] group, with a few important exceptions. Circulating concentrations of IL-4, IL-5, and IL-7 differed across the groups (*P* < 0.001 for all) and were significantly elevated in the G[+] and G[−] coinfected children, relative to both children with malaria-only (*P* < 0.001 for all) and healthy controls (*P* ≤ 0.024 for all), respectively (Figures 4(a), 4(b), and 4(c)). Consistent with our previous findings [40, 41, 43, 46], circulating IL-10 concentrations differed across the groups (*P* = 0.042; Figure 4(d)) and were higher in the* Pf*[+], G[−], and G[+] groups relative to health controls (*P* < 0.001, *P* = 0.038, and *P* = 0.035, resp.). However, the G[−] group had lower IL-10 levels compared to* Pf*[+] group (*P* = 0.016). Although there were no significant differences in the circulating IL-13 levels across the three infected groups after exclusion of the healthy controls from the initial omnibus K-W test (*P* = 0.366), IL-13 levels were generally suppressed in the infected children compared to the healthy controls, with the lowest levels being found in the G[+] coinfected group (Figure 4(e)). The TNF-*α* : IL-10 ratio also did not show intergroup significance (*P* = 0.329; Figure 4(f)), but a general skewing towards a type 2 inflammatory response was noted in the infected children relative to the healthy controls. Overall, we noted a greater cytokine response in the coinfected children, with higher concentrations of both pro- and anti-inflammatory cytokines. The cytokine profiles in coinfected children differed from those with monoinfection, which is an important consideration in the design and evaluation of host-biomarker-based diagnostics for infection. However, lower levels of a few key cytokines, such as TNF-*α* and IL-10, may be the key to controlling parasitemia without exacerbating anemia.

### 3.6. Multiple Mediation Model

**Figure 5:**
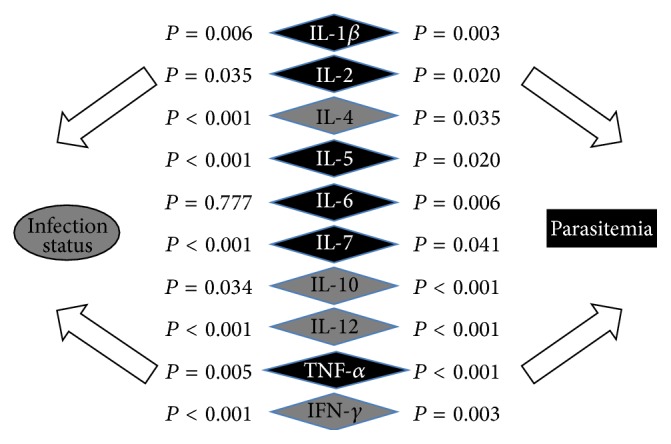
Multiple mediation model. Inflammatory mediators were correlated with the independent variable (infection status, *P* values to left of mediators) and parasitemia (*P* values to the right of mediators) individually, and those with significant correlations with the dependent variable (parasitemia) were used to populate the multiple mediation model. All nonparametric variables were log transformed. “infection status” was dummy coded as follows:* Pf*[+] monoinfected = 0; bacteremia coinfected = 1. The multiple mediation model was populated with ten inflammatory mediators that showed significant correlation with parasitemia, with age used as a covariate, and processed using 10,000 bootstrap samples. Post hoc pair-wise contrasts for the four significant mediators (black type; IL-4, IL-10, IL-12, and IFN-*γ*) were evaluated by examining the bootstrapped confidence intervals, with those intervals not including zero being significant.

Since we hypothesize that the relationship between infection status (independent variable) and parasitemia (dependent variable) is mediated, at least in part, through the production of cytokines, multiple mediation modeling was performed. As such, we first performed a Pearson correlation between each of the cytokines and parasitemia (Figure 5, *P* values right of cytokines) to select variables with significant correlations for populating the indirect.sbs SPSS macro. Parasitemia was significantly correlated with the following cytokines, in order of strength of association: IL-10, IL-12, TNF-*α*, IFN-*γ*, IL-1*β*, IL-6, IL-5, IL-2, IL-4, and IL-7. All of the cytokines were negatively associated with parasitemia, with the exception of IL-10, TNF-*α*, and IL-6. We also determined the correlation between the independent variable (infection status:* Pf*[+] only = 0 and bacteremia coinfected = 1) and the cytokines (Figure 5, *P* values left of cytokines). This revealed 13 mediators of inflammation that correlated with infection status, which were, in order of strength of significance, IL-4, IL-5, IL-7, IL-15, IFN-*α*, IL-12, IFN-*γ*, IL-17, IL-1Ra, TNF-*α*, IL-1*β*, IL-10, and IL-2. Of those, only TNF-*α* and IL-10 had negative associations with infection status. The remainder of the cytokines were positively associated with infection status, indicating that inflammatory mediator levels were greater in bacteremia coinfected children than those with* Pf*[+] monoinfection. The multiple mediation model was then processed with 10,000 bootstrap samples, which yielded four significant cytokines: IL-4, IL-10, IL-12, and IFN-*γ* (Figure 5: significant cytokines, black text; nonsignificant cytokines, white text). The bootstrapped paired contrasts from the analysis indicated that IL-4 had the greatest mediational effect between infection status and parasitemia.

## 4. Discussion

In the current study, we have characterized the inflammatory milieu of children residing in a holoendemic* P. falciparum* region that were monoinfected with malaria and coinfected with either G[+] or G[−] bacteremia. Our previous investigations in the area revealed that children who were either exposed (seropositive) or infected (proviral DNA positive) with HIV-1 had significant differences in their inflammatory mediator profiles than children with malaria alone [32]. Exclusion of HIV-1-exposed and HIV-1-positive children from the cytokines analyses demonstrated that children with either G[−] or G[+] organisms and falciparum malaria had reduced parasite burden and similar erythropoietic indices to children with malaria alone. This study provides insight into the changing dynamics of cytokine profiles in mono- and coinfected children, an important factor in furthering the understanding of immune mechanisms and host-pathogen biology in high-disease burden populations.

Although we previously discovered that malaria-infected children with G[−] organisms from this region had reduced parasitemia and enhanced risk of SMA in multivariate analyses controlling for known contributors of childhood anemia [5], differences in erythrocytic indices (e.g., Hb, Hct, RBC count, and SMA) did not differ in the children presented here in which cytokines were measured. The difference in erythrocytic indices between the two studies is likely due to the fact that all three groups of parasitemic children examined here were very ill, with exceedingly low levels of Hb, and, as such, significant differences between the groups were difficult to detect. This notion is supported by the data illustrating that the healthy control group had median Hb levels of 11.7 g/dL, while those in the* Pf*[+], and G[−], and G[+] coinfected groups were 5.9, 5.8, and 6.0 g/dL, respectively.

The only way to determine if this is indeed the case is to perform additional cytokine measurements on a larger group of children stratified according to the same parameters and include children that are not as severely anemic. We are currently completing longitudinal follow-up studies in a large cohort of children (*n* = 1,643) in the region and have future plans to perform additional studies to characterize the inflammatory milieu in coinfected children. However, it is important to note that severe anemia is common in malaria-infected children in the region, and additional coinfections often exacerbate the problem, making it difficult to find polymicrobial-infected individuals that are not severely anemic. One important erythrocytic finding was the decreased MCHC in coinfected children. This finding may indicate a microcytic hypochromic anemia, or, alternatively, iron deficiency anemia resulting from increased levels of proinflammatory cytokines in the coinfected groups compounded by poor nutritional status, a common phenomenon in the study area [11].

Consistent with previous studies [6, 47], NTS was the most prevalent bacterial species isolated from blood cultures in* Pf*[+]-coinfected children in the current study. Although the risk factors for this phenomenon include increased malnutrition, malarial anemia, unclean food and water, HIV-1 infection, contact with animals, age < 3 yrs, excessive iron availability from hemolysis and anemia, and sickle cell allele carriage (reviewed in [6]), coexistence of these factors in differing proportions appears the most plausible explanation of increased prevalence of NTS bacteremia in African children. In the cohort examined, there was a notable absence of typical childhood pathogens such as* S. pneumoniae* and* H. influenzae*, which may be partially explained by (a) vaccination efforts in Kenya (i.e.,* Haemophilus influenzae* type b [Hib]-conjugate vaccination began in November 2001); (b) the fastidiousness of these organisms; and (c) unprescribed antimicrobial treatment resulting in reduced pathogen recovery, particularly in individuals with low bioburdens. Our results showing a higher rate of* S. aureus* are consistent with studies elsewhere in Africa [10] and the spectrum of isolates reported here followed those of similar studies in the surrounding regions [9].

Although age did not differ in our clinical categories, prevalence of female gender was higher in both coinfected groups relative to the malaria-only group. The reason for this difference is unclear and requires further investigation. However, potential explanations could be related to differences in cultural and social practices of caregivers for females versus males and/or true physiological differences in the response to bacterial pathogens between the genders.

The most predominate clinical finding in our investigation was the reduced parasite burden in G[−] coinfected children with nearly fivefold lower median parasitemia and sixfold lower geometric mean parasitemia than the* Pf*[+] group. In addition, there was a significantly lower proportion of children with HDP in both the G[−] and G[+] coinfected groups, which was more pronounced in the presence of G[−] organisms. Greater reductions in all of the parasitemic parameters for the G[−] coinfected group, relative to those with G[+] pathogens, may reflect the greater inflammatogenic potential of lipopolysaccharide (LPS) versus the G[+] cell wall constituents [48]. Findings here showing lower parasite burdens in coinfected children in the absence of worsening anemia are similar to previous studies in Mozambique in which lower parasite densities, but not hematocrit, were observed in children with severe malaria and bacteremia [49].

Measurement of mediators of inflammation revealed that proinflammatory cytokines were highest in G[−] coinfected children that had the lowest parasitemia, followed by the G[+] coinfected group with slightly higher parasitemia, and lowest in the malaria monoinfected children with the highest parasitemia. The inverse association between parasitemia and elevated levels of IL-1*β*, IL-1Ra, IL-12, IL-15, IL-17, IFN-*γ*, and IFN-*α* in G[−] coinfected children, compared to malaria monoinfection, suggests that this profile may favor induction of antiparasitic activities. However, it remains unclear if IL-1*β* was responsible for controlling parasitemia, since the increase observed in the G[−] coinfected group may have been counterbalanced by a similar increase in IL-1Ra, as evidenced by similarities in the IL-1*β* : IL-1Ra ratio across the groups. The increase in IL-12 in the coinfected groups and decrease in malaria monoinfection are consistent with previous studies indicating suppression of IL-12 in children with malaria [23, 40]. Elevation of IL-12 in the coinfected groups in the context of lower parasitemia is consistent with previous human and murine studies showing that IL-12 is important for controlling parasitemia [50–52]. The upregulation of IL-15 in coinfected children is consistent with the potent type 1 immune activities of IL-15, which includes parasite and bacterial clearance, and induction of humoral responses [53]. Although previous* in vitro* studies using murine erythroid cells demonstrated that IL-15 enhances the erythropoiesis-stunting effect of IFN-*γ* [54], elevation of both IL-15 and IFN-*γ* in the G[−] group, in the absence of decreased erythropoietic indices, suggests that their elevation does not necessarily promote worsening anemia. Although IL-15 is known to induce production of IL-17 from CD4+ T cells [55], plasma IL-17 was suppressed in malaria monoinfected children and similar in the other groups. These findings are consistent with previous studies in Indian malaria patients showing reduced plasma IL-17 in mild malaria relative to healthy controls [56] and our previous study in the larger Kenyan cohort showing suppression of IL-17 in SMA patients compared to those with uncomplicated malaria and non-SMA [57]. Additional proinflammatory cytokine results showing elevated IFN-*γ* and IFN-*α* in coinfected children relative to the monoinfected and healthy controls are consistent with reduced parasite densities in these individuals. This premise is supported by previous malaria studies demonstrating that IFN-*γ* mediates clearance of plasmodial parasites, while IFN-*α* may contribute to parasite clearance through induction of effector molecules such as nitric oxide (NO) [58]. In contrast, reduced TNF-*α* levels in the coinfected children relative to the malaria monoinfected children may be related to counterregulatory activities of IFN-induced increased NO that downregulate nitric oxide synthase- (NOS-) inducing cytokines, such as TNF-*α*, through a feedback mechanism [59]. Taken together, these results indicate an upregulation of the type 1 immune response in coinfected children, with the exception of TNF-*α*, a cytokine that if overproduced can promote malarial anemia through enhanced erythrophagocytosis and dyserythropoiesis [38].

Examination of the anti-inflammatory cytokine profile revealed that IL-4, IL-5, and IL-7 levels were elevated in the G[−] and G[+] coinfected children, while IL-10 was lowest in the G[−] coinfected group, and IL-13 was not significantly different across the groups. Although IL-4 has not been previously reported in the coinfected groups examined in this study, a study by Cabantous et al. [60] in Malian children showed an elevation of IL-4 in SMA compared to those with uncomplicated malaria. While it appears that downregulation of the type 1 immune response may have exacerbated disease in the Malian children, elevated IL-4 levels in coinfected children point to counterregulatory activity of IL-4 on the potentially damaging type 1 response and the synergistic effect of IL-4 on EPO [61], IL-6 [62], and IL-12 [63] in promoting erythropoiesis. This hypothesis appears the most plausible since the type 1 response was too potent to be attenuated by IL-4.

Elevated IL-7 levels in coinfected children may be related to its activity in supporting erythropoiesis [64], hence protecting against a reduction of hemoglobin levels in the markedly potent proinflammatory environment observed in these children. Additional findings showing reduced IL-10 levels in coinfected children in the context of lower parasitemia are consistent with previous studies showing that the level of parasitemia closely reflects the IL-10 pattern [65, 66]. This is supported by previous studies showing that increased IL-10 secretion by CD4+ T cells inhibits parasite killing in malaria infection [45, 67]. In addition, our previous studies, and those of others, demonstrated poor recovery from malarial anemia in children with elevated IL-10 levels [43], largely through reduced IL-12 production [40, 68].

To place the large number of cytokines measured in this study into a framework that could explain their influence on mediating the effect between infection status and parasitemia, we employed multiple mediation modeling with bootstrapping. This particular method was selected since bootstrapping is a nonparametric statistic that does not violate assumptions of normality and is, therefore, more robust for small sample sizes. The modeling identified IL-4, IL-10, IL-12, and IFN-*γ* as the strongest mediators between infection status and parasitemia, with paired contrasts from the analysis indicating that IL-4 had the largest mediational effect. The Pearson correlations, used to load the mediation model, showed an inverse association between IL-4, IL-12, and IFN-*γ*, and parasitemia and a positive correlation between IL-10 and parasitemia. Taken together, these findings suggest that a combination of type 1 and type 2 responses is required to control parasitemia and that key differences in cytokines between malaria monoinfected and coinfected children may represent the optimal response to infection in which the pathogen is effectively controlled with minimal impact on hemoglobin levels. An alternative, but not mutually exclusive, hypothesis for lower levels of parasitemia in children with high levels of proinflammatory cytokines may be that such children develop clinical signs such as fever earlier and, therefore, present at the hospital sooner in less advanced stages of disease with lower parasitic burden.

## 5. Conclusion

Results presented here characterized the cytokine profile that predominates in malaria and bacteremia coinfected children from a holoendemic transmission area and the specific mediators of inflammation that are responsible for improved control of malaria parasitemia without worsening anemia. Our findings add to the understanding of the cytokine pathways involved in control and recovery from various infections, especially during coinfection, and suggest a prominent role for cytokines, such as IL-4, that have historically received less attention in human malaria. Dissecting the interplay between conserved and unique immune pathways in high-disease burden populations with multiple coinfections requires an improved understanding of host-pathogen biology. This paper represents a path towards the process of characterizing the extensive variability and effect of types 1 and 2 immune responses during coinfection.

## Figures and Tables

**Table 1:** Bacterial isolate distribution.

Organism	Isolates	Percent of isolates
Gram negative organisms		
*Acinetobacter *sp.	1	2.8
*Escherichia coli*	1	2.8
*Pseudomonas aeruginosa*	1	2.8
*Salmonella *ssp.* enterica ser. Arizonae*	1	2.8
*Salmonella *ssp.* enterica ser. Enteritidis*	2	5.6
*Salmonella *ssp.* enterica ser. Typhimurium*	16	44.4

Gram positive organisms		
*Enterococcus *sp.	1	2.8
*Staphylococcus aureus*	13	36.1

Organisms isolated from pediatric blood culture according to Gram reaction category were included in these analyses. Blood was drawn into pediatric Isostat tubes, plated onto Chocolate Agar plates, and incubated for 18 hrs at 37°C with 5% CO_2_. Subcultured colonies were incubated 18–24 hrs on inverted specialty media (Chocolate Agar, MacConkey's Agar, Mannitol Salt Agar, and Blood Agar plates). Plates were incubated for an additional four days if no growth was obtained in the first 24 hrs. Plates were inspected daily, and unique colonies were identified through standard microbiological and biochemical techniques (i.e., Gram stain, colony characteristics, API biochemical panels, and agglutination tests).

**Table 2:** Clinical, demographic, and hematological characteristics of the study participants.

Characteristic	Healthy controls	Parasitemic group	G[−] coinfection	G[+] coinfection	*P* ^*∗*^
Number of subjects	14	156	22	14	N/A
Age, months	7.07 (11.39)	9.08 (8.70)	7.38 (7.50)	12.11 (9.94)	0.161
Gender, male/female^*∗∗*^	8/6	88/68	6/16	5/9	**0.041**
Axillary temperature (°C)	36.4 (0.4)	37.5 (1.7)	38.0 (1.5)	37.1 (1.6)	0.141
Glucose (mMol/L)	4.89 (0.59)	4.90 (1.48)	5.59 (1.70)	5.10 (4.94)	0.067
WBC (×10^9^/*μ*L)	10.50 (3.95)	11.75 (6.95)	13.35 (9.15)	9.10 (4.60)	**0.047**
Lymphocytes (×10^3^/*μ*L)	6.70 (2.05)	5.60 (3.87)	6.65 (4.20)	4.80 (4.95)	0.431
Monocytes (×10^3^/*μ*L)	0.80 (0.55)	1.10 (0.90)	1.15 (0.67)	0.60 (0.90)	0.161
Granulocytes (×10^3^/*μ*L)	3.10 (2.35)	4.75 (4.17)	4.75 (5.80)	2.60 (2.30)	**0.010**
Hemoglobin (Hgb, g/dL)	11.7 (0.8)	5.9 (3.7)	5.8 (1.8)	6.0 (2.8)	0.594
Hematocrit (Hct, %)	35.2 (1.9)	18.8 (11.7)	19.1 (4.7)	19.7 (7.4)	0.792
RBC (×10^6^/*μ*L)	4.82 (0.31)	2.85 (1.90)	2.69 (1.27)	2.93 (1.09)	0.642
MCHC (g/dL)	33.4 (0.9)	32.5 (2.5)	30.9 (4.2)	30.9 (2.8)	**0.003**
RDW	16.4 (4.3)	21.2 (4.8)	22.4 (3.9)	22.7 (5.8)	0.053
Platelets (×10^3^/*μ*L)	355 (219)	153 (111)	159 (107)	160 (98)	0.720
SMA, Hb < 5.0 g/dL; *n* (%)^*∗∗*^	N/A	42 (26.9)	6 (27.3)	2 (14.3)	0.562
Mortality, *n* (%)^*∗∗∗*^	0 (0)	6 (3.8)	2 (9.1)	0 (0)	0.459

HIV-1 negative children (aged < 3 yrs) infected with *P. falciparum* malaria were divided into parasitemic (*Pf*[+]), G[−] bacteremia coinfected, and G[+] bacteremia coinfected groups. In addition, a cohort of afebrile healthy children without parasitemia and hemoglobin (Hb) > 11 g/dL were included as controls to detect depressed cytokine levels associated with infection. WBC: white blood cells; RBC: red blood cells; MCHC: mean corpuscular hemoglobin concentration; RDW: red cell distribution width. ^*∗*^Data are presented as median (interquartile range) and compared using the Kruskal-Wallis test unless stated otherwise. ^*∗∗*^Differences in categorical variables were compared using Pearson's *χ* ^2^ test. ^*∗∗∗*^Generalized Fisher's exact test. Complete blood counts were determined in venous blood using a Coulter AcT diff2 (Beckman Coulter Corp.).

## References

[1] WHO (2010). *World Malaria Report 2010*.

[2] McElroy P. D., Ter Kuile F. O., Hightower A. W. (2001). All-cause mortality among young children in western Kenya. VI: The Asembo Bay Cohort Project. *American Journal of Tropical Medicine and Hygiene*.

[3] Ong'echa J. M., Keller C. C., Were T. (2006). Parasitemia, anemia, and malarial anemia in infants and young children in a rural holoendemic *Plasmodium falciparum* transmission area. *American Journal of Tropical Medicine and Hygiene*.

[4] Otieno R. O., Ouma C., Ong'echa J. M. (2006). Increased severe anemia in HIV-1-exposed and HIV-1-positive infants and children during acute malaria. *AIDS*.

[5] Were T., Davenport G. C., Hittner J. B. (2011). Bacteremia in Kenyan children presenting with malaria. *Journal of Clinical Microbiology*.

[6] Morpeth S. C., Ramadhani H. O., Crump J. A. (2009). Invasive non-typhi *Salmonella* disease in Africa. *Clinical Infectious Diseases*.

[7] Friedland I. R. (1992). Bacteraemia in severely malnourished children. *Annals of Tropical Paediatrics*.

[8] Walsh A. L., Phiri A. J., Graham S. M., Molyneux E. M., Molyneux M. E. (2000). Bacteremia in febrile Malawian children: clinical and microbiologic features. *Pediatric Infectious Disease Journal*.

[9] Berkley J. A., Lowe B. S., Mwangi I. (2005). Bacteremia among children admitted to a rural hospital in Kenya. *The New England Journal of Medicine*.

[10] Sigaúque B., Roca A., Mandomando I. (2009). Community-acquired bacteremia among children admitted to a rural hospital in Mozambique. *Pediatric Infectious Disease Journal*.

[11] Bwibo N. O., Neumann C. G. (2003). The need for animal source foods by Kenyan children. *Journal of Nutrition*.

[12] Grau G. E., Taylor T. E., Molyneux M. E. (1989). Tumor necrosis factor and disease severity in children with falciparum malaria. *The New England Journal of Medicine*.

[13] Kwiatkowski D., Sambou I., Twumasi P. (1990). TNF concentration in fatal cerebral, non-fatal cerebral, and uncomplicated *Plasmodium falciparum* malaria. *The Lancet*.

[14] Perkins D. J., Were T., Davenport G. C., Kempaiah P., Hittner J. B., Ong'echa J. M. (2011). Severe malarial anemia: innate immunity and pathogenesis. *International Journal of Biological Sciences*.

[15] Ulloa L., Tracey K. J. (2005). The ‘cytokine profile’: a code for sepsis. *Trends in Molecular Medicine*.

[16] Bozza F. A., Salluh J. I., Japiassu A. M. (2007). Cytokine profiles as markers of disease severity in sepsis: a multiplex analysis. *Critical Care*.

[17] Schwarzer E., Skorokhod O. A., Barrera V., Arese P. (2008). Hemozoin and the human monocyte—a brief review of their interactions. *Parassitologia*.

[18] Cho S.-Y., Choi J.-H. (2014). Biomarkers of Sepsis. *Infection and Chemotherapy*.

[19] Kremsner P. G., Winkler S., Brandts C. (1995). Prediction of accelerated cure in *Plasmodium falciparum* malaria by the elevated capacity of tumor necrosis factor production. *American Journal of Tropical Medicine and Hygiene*.

[20] Luty A. J. F., Lell B., Schmidt-Ott R. (1999). Interferon-*γ* responses are associated with resistance to reinfection with *Plasmodium falciparum* in young African children. *The Journal of Infectious Diseases*.

[21] D'Ombrain M. C., Robinson L. J., Stanisic D. I. (2008). Association of early interferon-*γ* production with immunity to clinical malaria: a longitudinal study among Papua New Guinean children. *Clinical Infectious Diseases*.

[22] Day N. P. J., Hien T. T., Schollaardt T. (1999). The prognostic and pathophysiologic role of pro- and antiinflammatory cytokines in severe malaria. *Journal of Infectious Diseases*.

[23] Perkins D. J., Weinberg J. B., Kremsner P. G. (2000). Reduced interleukin-12 and transforming growth factor-*β*1 in severe childhood malaria: relationship of cytokine balance with disease severity. *Journal of Infectious Diseases*.

[24] Ouma C., Davenport G. C., Were T. (2008). Haplotypes of IL-10 promoter variants are associated with susceptibility to severe malarial anemia and functional changes in IL-10 production. *Human Genetics*.

[25] Awandare G. A., Hittner J. B., Kremsner P. G. (2006). Decreased circulating macrophage migration inhibitory factor (MIF) protein and blood mononuclear cell MIF transcripts in children with *Plasmodium falciparum* malaria. *Clinical Immunology*.

[26] Lavoie P. M., Huang Q., Jolette E. (2010). Profound lack of interleukin (IL)-12/IL-23p40 in neonates born early in gestation is associated with an increased risk of sepsis. *Journal of Infectious Diseases*.

[27] Marchant A., Goldman M., Devière J., Byl B., Vincent J. L., Groote D. D. (1994). Interleukin-10 production during septicaemia. *The Lancet*.

[28] Suárez-Santamaría M., Santolaria F., Pérez-Ramírez A. (2010). Prognostic value of inflammatory markers (notably cytokines and procalcitonin), nutritional assessment, and organ function in patients with sepsis. *European Cytokine Network*.

[29] Ma X., Sun J., Papasavvas E. (2000). Inhibition of IL-12 production in human monocyte-derived macrophages by TNF. *The Journal of Immunology*.

[30] Giroir B. P. (1993). Mediators of septic shock: new approaches for interrupting the endogenous inflammatory cascade. *Critical Care Medicine*.

[31] Beier J. C., Oster C. N., Onyango F. K. (1994). *Plasmodium falciparum* incidence relative to entomologic inoculation rates at a site proposed for testing malaria vaccines in western Kenya. *The American Journal of Tropical Medicine and Hygiene*.

[32] Davenport G. C., Ouma C., Hittner J. B. (2010). Hematological predictors of increased severe anemia in Kenyan children coinfected with *Plasmodium falciparum* and HIV-1. *American Journal of Hematology*.

[33] WHO (2000). Severe falciparum malaria. World Health Organization, Communicable Diseases Cluster. *Transactions of the Royal Society of Tropical Medicine and Hygiene*.

[34] Were T., Ouma C., Otieno R. O. (2006). Suppression of RANTES in children with *Plasmodium falciparum* malaria. *Haematologica*.

[35] Preacher K. J., Hayes A. F. (2008). Asymptotic and resampling strategies for assessing and comparing indirect effects in multiple mediator models. *Behavior Research Methods*.

[36] Wilmanski J., Villanueva E., Deitch E. A., Spolarics Z. (2007). Glucose-6-phosphate dehydrogenase deficiency and the inflammatory response to endotoxin and polymicrobial sepsis. *Critical Care Medicine*.

[37] Williams T. N., Uyoga S., Macharia A. (2009). Bacteraemia in Kenyan children with sickle-cell anaemia: a retrospective cohort and case-control study. *The Lancet*.

[38] Clark I. A., Alleva L. M., Budd A. C., Cowden W. B. (2008). Understanding the role of inflammatory cytokines in malaria and related diseases. *Travel Medicine and Infectious Disease*.

[39] Luty A. J. F., Perkins D. J., Lell B. (2000). Low interleukin-12 activity in severe Plasmodium falciparum malaria. *Infection and Immunity*.

[40] Keller C. C., Yamo O., Ouma C. (2006). Acquisition of hemozoin by monocytes down-regulates interleukin-12 p40 (IL-12p40) transcripts and circulating IL-12p70 through an IL-10-dependent mechanism: in vivo and in vitro findings in severe malarial anemia. *Infection and Immunity*.

[41] Keller C. C., Davenport G. C., Dickman K. R. (2006). Suppression of prostaglandin E2 by malaria parasite products and antipyretics promotes overproduction of tumor necrosis factor-*α*: association with the pathogenesis of childhood malarial anemia. *Journal of Infectious Diseases*.

[42] Awandare G. A., Ouma Y., Ouma C. (2007). Role of monocyte-acquired hemozoin in suppression of macrophage migration inhibitory factor in children with severe malarial anemia. *Infection and Immunity*.

[43] Ong'echa J. M., Remo A. M., Kristoff J. (2008). Increased circulating interleukin (IL)-23 in children with malarial anemia: in vivo and in vitro relationship with co-regulatory cytokines IL-12 and IL-10. *Clinical Immunology*.

[44] Lyke K. E., Burges R., Cissoko Y. (2004). Serum levels of the proinflammatory cytokines interleukin-1 beta (IL-1*β*), IL-6, IL-8, IL-10, tumor necrosis factor alpha, and IL-12(p70) in Malian children with severe *Plasmodium falciparum* malaria and matched uncomplicated malaria or healthy controls. *Infection and Immunity*.

[45] Couper K. N., Blount D. G., Wilson M. S. (2008). IL-10 from CD4+CD25-Foxp3-CD127—adaptive regulatory T cells modulates parasite clearance and pathology during malaria infection. *PLoS Pathogens*.

[46] Perkins D. J., Moore J. M., Otieno J. (2003). In vivo acquisition of hemozoin by placental blood mononuclear cells suppresses PGE_2_, TNF-*α*, and IL-10. *Biochemical and Biophysical Research Communications*.

[47] Graham S. M., Molyneux E. M., Walsh A. L., Cheesbrough J. S., Molyneux M. E., Hart C. A. (2000). Nontyphoidal *Salmonella* infections of children in tropical Africa. *Pediatric Infectious Disease Journal*.

[48] Hessle C. C., Andersson B., Wold A. E. (2005). Gram-positive and Gram-negative bacteria elicit different patterns of pro-inflammatory cytokines in human monocytes. *Cytokine*.

[49] Bassat Q., Guinovart C., Sigaúque B. (2009). Severe malaria and concomitant bacteraemia in children admitted to a rural Mozambican hospital. *Tropical Medicine and International Health*.

[50] Stevenson M. M., Tam M. F., Wolf S. F., Sher A. (1995). IL-12-induced protection against blood-stage *Plasmodium chabaudi* AS requires IFN-*γ* and TNF-*α* and occurs via a nitric oxide-dependent mechanism. *The Journal of Immunology*.

[51] Boutlis C. S., Lagog M., Chaisavaneeyakorn S. (2003). Plasma interleukin-12 in malaria-tolerant papua new guineans: inverse correlation with *Plasmodium falciparum* parasitemia and peripheral blood mononuclear cell nitric oxide synthase activity. *Infection and Immunity*.

[52] Bakir H. Y., Tomiyama C., Abo T. (2011). Cytokine profile of murine malaria: stage-related production of inflammatory and anti-inflammatory cytokines. *Biomedical Research*.

[53] Ing R., Gros P., Stevenson M. M. (2005). Interleukin-15 enhances innate and adaptive immune responses to blood-stage malaria infection in mice. *Infection and Immunity*.

[54] Means R. T., Krantz S. B., Luna J., Marsters S. A., Ashkenazi A. (1994). Inhibition of murine erythroid colony formation in vitro by interferon gamma and correction by interferon receptor immunoadhesin. *Blood*.

[55] Ziolkowska M., Koc A., Luszczykiewicz G. (2000). High levels of IL-17 in rheumatoid arthritis patients: IL-15 triggers in vitro IL-17 production via cyclosporin A-sensitive mechanism. *Journal of Immunology*.

[56] Jain V., Armah H. B., Tongren J. E. (2008). Plasma IP-10, apoptotic and angiogenic factors associated with fatal cerebral malaria in India. *Malaria Journal*.

[57] Ong'echa J. M., Davenport G. C., Vulule J. M., Hittner J. B., Perkins D. J. (2011). Identification of inflammatory biomarkers for pediatric malarial: anemia severity using novel statistical methods. *Infection and Immunity*.

[58] Kremsner P. G., Winkler S., Wildling E. (1996). High plasma levels of nitrogen oxides are associated with severe disease and correlate with rapid parasitological and clinical cure in *Plasmodium falciparum* malaria. *Transactions of the Royal Society of Tropical Medicine and Hygiene*.

[59] Florquin S., Amraoui Z., Dubois C., Decuyper J., Goldman M. (1994). The protective role of endogenously synthesized nitric oxide in staphylococcal enterotoxin B-induced shock in mice. *Journal of Experimental Medicine*.

[60] Cabantous S., Poudiougou B., Oumar A. A. (2009). Genetic evidence for the aggravation of plasmodium falciparum malaria by interleukin 4. *Journal of Infectious Diseases*.

[61] Peschel C., Paul W. E., Ohara J., Green I. (1987). Effects of B cell stimulatory factor-1/interleukin 4 on hematopoietic progenitor cells. *Blood*.

[62] Rennick D., Jackson J., Yang G., Wideman J., Lee F., Hudak S. (1989). Interleukin-6 interacts with interleukin-4 and other hematopoietic growth factors to selectively enhance the growth of megakaryocytic, erythroid, myeloid, and multipotential progenitor cells. *Blood*.

[63] Dybedal I., Larsen S., Jacobsen S. E. W. (1995). IL-12 directly enhances in vitro murine erythropoiesis in combination with IL-4 and stem cell factor. *The Journal of Immunology*.

[64] Aiello F. B., Keller J. R., Klarmann K. D., Dranoff G., Mazzucchelli R., Durum S. K. (2007). IL-7 induces myelopoiesis and erythropoiesis. *Journal of Immunology*.

[65] Othoro C., Lal A. A., Nahlen B., Koech D., Orago A. S. S., Udhayakumar V. (1999). A low interleukin-10 tumor necrosis factor-*α* ratio is associated with malaria anemia in children residing in a holoendemic malaria region in western Kenya. *Journal of Infectious Diseases*.

[66] Ageely H. M., Dawoud H. A., Heiba A. A. (2008). Anemia, interleukin-10, tumor necrosis factor alpha, and erythropoietin levels in children with acute, complicated and uncomplicated malignant malaria in Jazan, Saudi Arabia. *Journal of the Egyptian Society of Parasitology*.

[67] Hugosson E., Montgomery S. M., Premji Z., Troye-Blomberg M., Björkman A. (2004). Higher IL-10 levels are associated with less effective clearance of *Plasmodium falciparum* parasites. *Parasite Immunology*.

[68] Mohan K., Stevenson M. M. (1998). Interleukin-12 corrects severe anemia during blood-stage *Plasmodium chabaudi* AS in susceptible A/J mice. *Experimental Hematology*.

